# Decrease in Urinary Tract Infections Following Switch From Anti-Interleukin-6 Monoclonal Antibody to Ravulizumab in a Patient With Neuromyelitis Optica Spectrum Disorder (NMOSD): A Case Report

**DOI:** 10.7759/cureus.92891

**Published:** 2025-09-21

**Authors:** Seiya Takahashi, Taro Yasumoto, Akinori Futamura, Ryuta Kinno

**Affiliations:** 1 Neurology, Showa Medical University, Yokohama, JPN

**Keywords:** neuromyelitis optica spectrum disorder, ravulizumab, satralizumab, switching therapy, urinary tract infections

## Abstract

Satralizumab is an anti-interleukin-6 (IL-6) monoclonal antibody used for the treatment of neuromyelitis optica spectrum disorder (NMOSD). Although mild urinary tract infections (UTIs) or respiratory tract infections during immunotherapy may appear benign, they can progress to serious infections such as sepsis. We report a case of a 65-year-old woman with NMOSD who is currently under regular follow-up at our hospital and receiving biologic therapy. She was diagnosed with anti-aquaporin 4 antibody-positive NMOSD at the age of 53 and experienced multiple relapses thereafter. Approximately 10 years after disease onset, satralizumab was initiated, after which she developed recurrent UTIs. After switching to ravulizumab, the incidence of UTIs was halved despite no significant changes in prednisolone dose, HbA1c level, or urinary glucose level. The clinical course suggested a close association between satralizumab treatment and recurrent UTIs. This case highlights the potential increased susceptibility to recurrent UTIs associated with anti-IL-6 monoclonal antibody therapy. Clinicians should monitor for recurrent UTIs in NMOSD patients treated with satralizumab and consider alternative treatments, such as C5 complement inhibitors, if infections persist.

## Introduction

Satralizumab is an anti-interleukin (IL)-6 monoclonal antibody that targets the IL-6 receptor and is used for the treatment of neuromyelitis optica spectrum disorder (NMOSD) [1]. IL-6 plays a role in prolonging survival and enhancing anti-aquaporin (AQP) 4 antibody secretion by plasmablasts [2]. Satralizumab blocks the IL-6 signaling pathway associated with inflammation by inhibiting the binding of IL-6 to its receptor. Although infection-related adverse events have been established to exhibit a comparable prevalence in both satralizumab and placebo groups [3], it should be noted that in patients receiving immunotherapy, conditions that appear to be mild urinary tract infections (UTIs) or respiratory tract infections may develop into more serious infections, such as sepsis.

The complement inhibitor ravulizumab binds with high affinity to complement component C5, preventing its cleavage and thereby suppressing the formation of the membrane attack complex to reduce NMOSD relapses [4]. While C5 inhibition potently suppresses terminal complement activity, proximal complement functions, including C3 activation, C3b-mediated opsonization, and immune complex clearance, are preserved [5]. However, C5 complement inhibitors increase the risk of meningococcal infections, and therefore vaccination prior to administration, prophylactic antibiotics, and prompt antimicrobial therapy in cases of suspected meningococcal infection are recommended.

Here, we report a case of NMOSD in which recurrent UTIs developed after satralizumab therapy and subsequently improved following a switch to ravulizumab, a switch considered based on their distinct mechanisms of action. This case provides insight into the potential association between anti-IL-6 monoclonal antibodies and recurrent UTIs.

## Case presentation

**Figure 1:**
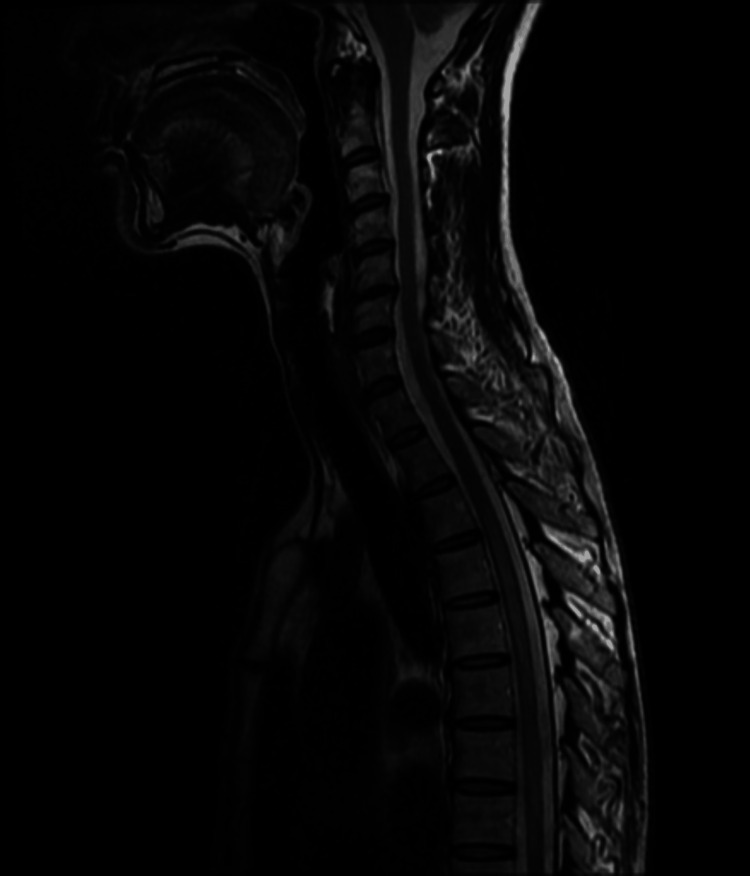
Spinal MRI at diagnosis (T2-weighted sagittal image) A longitudinal spinal cord lesion extending below the third thoracic level was observed.

A 65-year-old woman with anti-AQP4 antibody-positive NMOSD has been followed regularly at our hospital and treated with biologic therapies. She initially developed left-eye visual loss at the age of 53 and was diagnosed with left optic neuritis. Six months after onset, she experienced numbness in the thoracic spinal region and received steroid pulse therapy at a previous hospital before being referred to our institution. A few months later, her generalized numbness in the thoracic region worsened, prompting admission to our hospital. Upon admission, she had decreased visual acuity in the left eye and a left superior outer visual field defect. Muscle weakness was observed in the bilateral deltoids, iliopsoas, quadriceps, and hamstrings (manual muscle test, grade 4). She had decreased sensation over the entire thoracic area and hyperdeep tendon reflexes in the arms and legs, with a positive Babinski reflex. Blood tests were positive for anti-AQP4 antibody, and spinal magnetic resonance imaging (MRI) revealed a lesion extending over six vertebral segments (Figure 1). Cerebrospinal fluid examination revealed a high IgG index (2.1) with a normal cell count (1 /μL) and protein level (29 mg/dL). Oligoclonal bands were not observed (Table 1). Based on these findings, she was diagnosed with anti-AQP4 antibody-positive NMOSD.

**Table 1:** Summary of laboratory data at diagnosis The patient was positive for anti-AQP4 antibodies, and blood tests showed no findings suggestive of systemic autoimmune disease. Cerebrospinal fluid (CSF) analysis revealed near-normal cell count, protein, and glucose levels, but the IgG index was elevated and myelin basic protein was mildly increased, suggesting intrathecal IgG synthesis and demyelination. Oligoclonal bands were negative. ANA: antinuclear antibody; Anti-SS-A: anti-Sjögren’s syndrome-related antigen A antibody; Anti-SS-B: anti-Sjögren’s syndrome-related antigen B antibody; TSH: thyroid-stimulating hormone; FT3: free triiodothyronine; FT4: free thyroxine; Anti-TPO antibody: anti-thyroid peroxidase antibody; Anti-AQP4 antibody: anti-aquaporin-4 antibody

Category	Test	Result	Reference Range
Blood	Anti-SS-A antibody (U/mL)	2	<2-9.9
	Anti-SS-B antibody (U/mL)	2	<2-9.9
	ANA (titer)	<40	0-39
	TSH (μIU/mL)	0.29	0.3-4.5
	FT3 (pg/mL)	2.03	2-4.5
	FT4 (ng/dL)	0.87	0.7-1.8
	Anti-TPO antibody (IU/mL)	-	<5-15.9
	Anti-AQP4 antibody	Positive	-
CSF	Appearance	Clear	-
	Color	Colorless	-
	Cell count (cells/μL)	1	<5
	Protein (mg/dL)	29	10-40
	Glucose (mg/dL)	74	50-75
	Myelin basic protein (pg/mL)	100	<102
Others	IgG index	2.1	0.3-0.7
	Oligoclonal bands	Negative	0-1 bands = Negative, ≥2 bands = Positive

After the diagnosis, oral prednisolone (40 mg/day) and azathioprine (100 mg/day) were initiated. However, due to leukocyte count reduction, azathioprine was changed to tacrolimus (3 mg/day). Steroid reduction was attempted; however, the patient experienced several episodes of myelitis, prompting treatment with tacrolimus and intravenous immunoglobulin (0.4 g/kg/day for five days), and reduction of the dose of prednisolone to 10 mg/day. Nevertheless, the patient developed numerous lumbar compression fractures and steroid-induced diabetes. In addition, self-catheterization was required due to a neurogenic bladder. Thereafter, it was not possible to reduce prednisolone to ≤10 mg/day.

**Figure 2:**
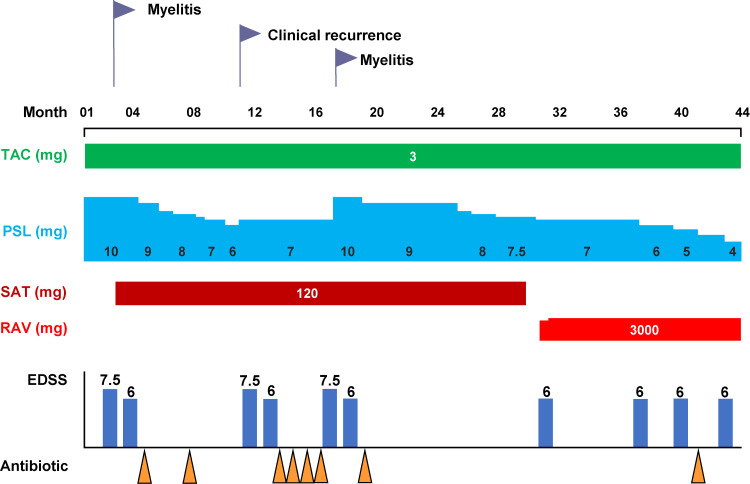
Clinical course The timing of each therapy, EDSS scores, and urinary white blood cell counts are shown. The clinical course is described in months, with disease relapses before the introduction of satralizumab defined as month 0. After the initiation of satralizumab, the patient experienced two relapses, leading to a switch to ravulizumab. The urinary white blood cell count decreased after switching from satralizumab to ravulizumab. EDSS: Expanded Disability Status Scale; PSL: prednisolone; RAV: ravulizumab; SAT: satralizumab; TAC: tacrolimus

Approximately 10 years after disease onset (at 63 years old), myelitis recurred with 10 mg/day of prednisolone; therefore, satralizumab was introduced, and the dose of prednisolone was reduced by 0.5 mg/month (Figure 2). One year after the initiation of satralizumab and after receiving coronavirus disease 2019 vaccination, the patient experienced a generalized area of numbness in the thoracic region and weakness in both lower limbs, which were considered manifestations of NMOSD recurrence. Although severe spinal cord atrophy on MRI made it difficult to confirm new lesions, the persistent clinical symptoms led us to conclude that this was a relapse of myelitis.

The patient received intravenous methylprednisolone (1 g/day for five days), and the dose of prednisolone was increased to 10 mg/day. During the satralizumab treatment period, the prednisolone dose was reduced to 7.5 mg/day. However, the patient experienced recurrent UTIs once every four months. Levofloxacin was effective in the first three episodes of UTI, and UTIs recurred monthly thereafter. Urine cultures collected during the fifth episode revealed levofloxacin-resistant *Escherichia coli*, and sulfamethoxazole-trimethoprim was administered. However, six months later, UTI recurred, prompting a change in treatment to cephalexin. Retrospective review of medical records showed no episode of UTI prior to satralizumab initiation. The patient denied any antibiotic treatment for UTIs prescribed at other hospitals.

We switched to ravulizumab therapy because we considered that satralizumab was not only ineffective for NMOSD but may also affect the development of UTI. After switching to ravulizumab, the dose of prednisolone was reduced to 4 mg/day. Eight months after switching to ravulizumab, only one episode of UTI occurred, which was treated with cephalexin. The incidence of UTI was halved during the ravulizumab treatment period compared to the satralizumab treatment period, despite no apparent difference in prednisolone dose, HbA1c level, and urinary glucose level (Table 2). Based on these clinical courses, we considered that the recurrent UTIs were closely associated with satralizumab treatment.

**Table 2:** Urinary examination The number of urinary tract infections (UTIs) was defined by the presence of bladder irritation symptoms and urinalysis findings, and counted as the number of antibiotic treatments administered. ^†^ Urine bacteria were evaluated microscopically on urine specimens, and those with more than half of the field of view observed under the microscope were determined as positive results. ^‡^ Urine glucose evaluates the amount of glucose in the urine, with more than 100 mg/dL being determined as a positive result. Note the significant decrease in the number of UTIs after RAV therapy. PSL: prednisolone; RAV: ravulizumab; SAT: satralizumab

	SAT	RAV
No. of UTI (times per six months)	0.86	0.43
PSL dose (mg per day)	8.3	5.9
HbA1c (%) (normal range: 4.6-6.2%)	5.9 ± 0.2	6.0 ± 0.1
No. of urine examination (times)	27	7
Positive urine bacteria (%)^†^ (positive if >50% of field of view under microscopy)	92.6	71.4
Positive urine glucose (%)^‡^ (positive if >100 mg/dL)	3.7	0

## Discussion

Our patient had NMOSD and developed recurrent UTIs after satralizumab therapy, which resolved after switching to ravulizumab therapy (Figure 1). A previous study has reported that UTIs do not increase with less than 10 mg of steroids or a cumulative dose under 700 mg [6]. Moreover, it is known that the risk of UTIs increases with higher HbA1c, but there is no significant difference in UTI risk between patients with HbA1c <6% and those with HbA1c 6-7% [7]. Because the steroid dose was less than 10 mg and the lower HbA1c level throughout the duration of both treatments (Table 1), the differences in UTI frequency could not be fully explained by the differences in steroid dose or HbA1c in our case. Recurrent UTIs are defined as two or more in six months (or three or more in one year) [8]. Our case met both definitions after receiving satralizumab treatment, whereas there was no apparent recurrence of UTIs after switching to ravulizumab treatment. Taking these findings together, we consider satralizumab therapy to be the most critical factor affecting the recurrent UTI in this case.

The most common cause of UTIs (80%) is uropathogenic *E. coli *(UPEC) [9]. Upon recognition of UPEC via toll-like receptor 4 in the bladder epithelium, IL-6 and IL-8 are secreted from bladder epithelial cells [9]. The binding of IL-6 to its receptor causes activation of JAK, which in turn activates Stat3 to regulate transcription of targets, including antimicrobial peptides (AMPs) [10]. AMPs promote bactericidal, bacteriostatic, and immunomodulatory actions [11]. Indeed, a previous study has shown that following acute cystitis, IL-6 knockout mice have significantly increased urinary bacteria compared with wild-type mice, and administration of IL-6 into the bladder of these mice reduced intracellular bacterial colonies [10]. Based on the course of this case, the use of an anti-IL-6 monoclonal antibody may inhibit IL-6/Stat3 signaling and weaken the defense mechanism against UTIs. Additionally, tocilizumab, which also inhibits IL-6, completely suppresses C-reactive protein production and may delay the diagnosis of severe infections [12]. Delayed diagnosis of UTIs can lead to progression to acute pyelonephritis, and antibiotic treatment for recurrent UTIs may lead to the emergence of resistant bacteria. The overall mortality rate for acute pyelonephritis has been reported to be approximately 7.4% in Hong Kong [13], and immunosuppression in patients with acute pyelonephritis is an independent predictor of mortality [14].

Ravulizumab, a complement inhibitor, potently suppresses terminal complement activity by inhibiting C5; however, proximal complement functions, including C3 activation, C3b-mediated opsonization, and immune complex clearance, are preserved [5]. Nevertheless, UPEC secretes a protein involved in colonization (Pic), which cleaves proximal complement factors, including C3, and has been reported to increase the risk of bacteremia and sepsis [15]. This indicates that proximal complement plays a critical role in host defense against *E. coli*. Therefore, in the present case, switching to ravulizumab was considered, taking into account the preservation of proximal complement function. When recurrent UTIs occur during treatment with anti-IL-6 monoclonal antibody agents, switching to other biologic therapies, as in this case, should be considered. Nonetheless, as this report describes a single case, further studies using experimental models and larger patient cohorts are needed to clarify the relationship between anti-IL-6 therapy and infection risk.

## Conclusions

This case demonstrated that a patient with NMOSD developed recurrent UTIs after initiating satralizumab treatment, which improved following a switch to ravulizumab. Inhibition of IL-6 signaling may impair immune defense against UTIs, and therefore, careful monitoring for the occurrence and recurrence of UTIs is necessary during anti-IL-6 monoclonal antibody therapy. In particular, when recurrent UTIs are observed, early consideration of switching to C5 complement inhibitors is advisable to prevent worsening infections and the emergence of resistant bacteria. This case provides an important cautionary message for clinicians treating NMOSD patients with satralizumab, highlighting the need for vigilance in infection management during treatment.
